# Effect of lithium ions on cementoblasts in the presence of lipopolysaccharide *in vitro*

**DOI:** 10.3892/etm.2015.2276

**Published:** 2015-02-09

**Authors:** SHANG GAO, YUZHUO WANG, XIAOLONG WANG, PENG LIN, MIN HU

**Affiliations:** Department of Orthodontics, School of Stomatology, Jilin University, Changchun, Jilin 130021, P.R. China

**Keywords:** periodontitis, cementum, lithium

## Abstract

The applications of lithium ions as an agent to facilitate bone formation have been widely documented; however, the effect of lithium ions in the periodontitis model has not yet been elucidated. The aim of the present study, therefore, was to investigate the effect of single lithium ions in the presence of lipopolysaccharide (LPS). A periodontitis model was induced in cementoblasts using LPS. The cytotoxic effect of the lithium ions on the cementoblasts was studied through the MTT assay. Alkaline phosphatase analysis and alizarin red staining were performed to investigate the effect of the lithium ions on differentiation. To examine the effect of lithium ions on osteoclastogenesis, osteoprotegerin (OPG) and receptor activator of nuclear factor-κB ligand (RANKL) mRNA and protein expression levels were assessed using reverse transcription-polymerase chain reaction analysis and ELISA, respectively. Compared with the effect induced by lithium ions on normal cementoblasts, proliferation and differentiation were downregulated following the co-incubation of the cementoblasts with LPS and lithium ions. Furthermore, the lithium ions appeared to alter osteoclastogenesis by regulating the OPG/RANKL ratio. In conclusion, the present findings suggest that lithium ions can downregulate proliferation and differentiation in a periodontitis model. Further studies should be undertaken prior to the acceptance of lithium ions for use in the clinic.

## Introduction

Characterized by gingival inflammation and periodontal tissue impairment, periodontitis is a serious threat to the oral health of human beings. In general, patients with periodontitis suffer from loose teeth due to the bone resorption without experiencing any pain (1,2). Over the past decades, a number of therapeutic approaches have been actively developed to prevent and treat periodontitis, and one of these potential treatment strategies is to facilitate bone regeneration via the usage of synthetic scaffolds. With their desirable osteoconductivity, bioactivity, interconnected porosity and biocompatibility, several well-prepared scaffolds have been generated and applied to maintain, induce and restore biological functions (3,4). As a consequence, studies into the development of novel scaffolds and associated investigations into the bio-effects of these scaffolds on disease models are intensively pursued in the biomedical field.

As a crucial structure in periodontal tissue, the cementum connects with the alveolar bone via fibers, retains the position of the teeth and shares a similar composition to bone; however, under conditions of impairment, the cementum typically undergoes no or little remodeling due to the high content of inorganic salt in its structure (5,6). To regenerate periodontal tissue, cementogenesis has been considered to be an undividable route that is regulated via various signals (7). According to previous studies, wnt signaling may participate in the formation of the root cementum and is involved in the differentiation and proliferation of cementoblasts (8–11).

By inhibiting the β-catenin inhibitor glycogen synthase kinase 3β, lithium ions can activate wnt signals *in vitro* and *in vivo* (3). Upon a suitable incubation concentration, lithium ions can enhance the proliferation of human mesenchymal stem cells, resulting in the formation of osteogenic and adipogenic lineages (12,13). Furthermore, a suitable combination of calcium phosphate core and lithium coating can promote the proliferation of MG63 cells and enhance the protein expression of osteogenic transcription factors (14,15). In addition to the above *in vitro* evidence, the high osteoconductivity of lithium ions has been equally documented *in vivo*, and lithium ions have been demonstrated to enhance bone repair and facilitate bone regeneration in a murine model (16–18). Lithium ions have been considered as a possible approach to improve dental implant osseointegration by facilitating osteoblast differentiation (19,20). More notably, lithium-doped bioglass has been shown to significantly enhance the proliferation and differentiation of periodontal ligament cells upon the activation of wnt signals (20). In combination, these findings suggest the potential of lithium-based materials as agents for bone-related disease, including certain diseases in the field of dentistry; however, to the best of our knowledge, the aforementioned studies were only carried out in natural physical conditions rather than in inflammatory environments. It is therefore necessary to explore the effect of lithium-based materials on a disease model, such as periodontitis. As such, the aim of this study was to investigate the effect of lithium chloride (LiCl) on cementoblast function, particularly in the presence of lipopolysaccharide (LPS).

## Materials and methods

### Preparation of LPS

LPS was purified from *Prophyromonas gingivalis* (ATCC 33277; American Type Culture Collection, Manassas, VA, USA) using the cold MgCl_2_/ethanol procedure. The prepared LPS was suspended in LPS-free water at various experimental concentrations for further use.

### Cell culture

An immortalized murine cementoblast cell line (OCCM-30), which was a gift from Professor Somerman at the University of Washington (Seattle, WA, USA), was cultured in accordance with previously described methods (21). The OCCM-30 cells were maintained in Dulbecco’s Modified Eagle’s Medium/F12 (Invitrogen Life Technologies, Carlsbad, CA, USA), supplemented with 10% fetal bovine serum containing 100 U/ml penicillin and 100 μg/ml streptomycin in a humidified atmosphere of 5% CO_2_ at 37°C.

### Cytotoxicity studies of LPS and LiCl

MTT assays were carried out to quantify the cytotoxicity of LPS and LiCl (Sigma-Aldrich Trading Co., Ltd., Shanghai, China). In a typical procedure, OCCM-30 cells were cultured in 96-well plates at a density of 5×10^3^ per well for 12 h to allow the attachment of the cells. Serial dilutions of LPS and LiCl were then added to separate culture media. At the end of various incubation periods, the media containing LPS and LiCl were removed, and the cells were treated with MTT for a further 4 h. Following MTT treatment, the supernatant was removed and the formazan crystals were dissolved by the addition of dimethylsulfoxide (DMSO; MP Biomedicals, LLC, Santa Ana, CA, USA). The absorbance at 490 nm was measured via a micro-ELISA reader (iMark 680; Bio-Rad Laboratories, Inc., Hercules, CA, USA). Six replicates were performed for each group and the percentage viability was normalized to the viability of untreated cells.

### Cytotoxicity studies of LiCl in a periodontitis model

The periodontitis model was prepared using LPS as the inducing agent, according to a previous route (22). An MTT assay was carried out to quantify the cytotoxicity of LiCl in the presence of LPS (25 ng/ml). OCCM-30 cells were cultured in 96-well plates at a density of 5×10^3^ per well for 12 h to allow the attachment of the cells. A total of 50 μg/ml LiCl was then added to the culture medium. At the end of the incubation period, the medium containing LiCl was removed, and the cells were treated with MTT for further 4 h. The supernatant was then removed and the formazan crystals were dissolved by the addition of DMSO. The absorbance at 490 nm was measured via a micro-ELISA reader. Six replicates were performed for each group and the percentage viability was normalized to the viability of untreated cells.

### Examination of alkaline phosphatase (ALP) activity

OCCM-30 cells at a density of 2×10^4^ were cultured overnight in a 24-well plate to allow the attachment of the cells. Following culture, the cells were washed twice with phosphate-buffered saline (PBS) for 10 sec and then divided into four treatment groups, as follows: i) Blank group (no additional agent); ii) LiCl group (treatment with 50 μg/ml LiCl); iii) LPS group (treatment with 25 ng/ml LPS); and iv) LiCl + LPS group (treatment with 50 μg/ml LiCl and 25 ng/ml LPS). The three experimental groups were treated for one week when ALP expression was prominent. After seven days of incubation, 0.9% NaCl solution containing 1% Triton X-100 (Roche Diagnostics, Basel, Swirzerland) was selected to dissolve out the cellular proteins. Centrifugation was performed at 150 × g for 4 min at room temperature and the supernatants were further assayed for ALP activity using an ALP assay kit (Sigma-Aldrich, St. Louis, MO, USA). All results were normalized to the total protein levels in the OCCM-30 cells.

### Mineralization assay with alizarin red staining

OCCM-30 cells at a density of 1×10^5^ were cultured in a 24-well plate. Two days later, the medium was removed and the cells were washed twice with PBS for 10 sec. The cells (control/LiCl/LPS/LiCl+LPS) were then cultured in osteogenic medium [5 nM dexamethasone, 250 μM L-ascorbic acid 2-phosphate, 10 mM β-glycerophosphate (Sigam-Aldrich) in DMEM/F12] at 37°C. After 10 days of incubation, the cells were washed with deionized water several times and fixed in ice-cold 95% ethanol at room temperature for 30 min. The cells were then stained with alizarin red (2%, pH 4.2) for 15 min and observed under an Olympus BX-51 optical system microscope (Olympus Corp., Tokyo, Japan) under white light. To obtain quantified analysis, cetylpyridinium chloride (1%, 1 ml; Shenggong Co., Ltd., Shanghai, China) was added to the plates, and the optical density (OD) values were measured at 540 nm using a micro ELISA reader (iMark 680; Bio-Rad Laboratories, Inc.).

### RNA isolation and reverse transcription-polymerase chain reaction (RT-PCR)

To determine the levels of mRNA expression, OCCM-30 cells at a density of 5×10^4^ were cultured in a six-well plate. After 12 h, the three experimental groups (50 μg/ml LiCl, 25 ng/ml LPS and 50 μg/ml LiCl + 25 ng/ml LPS) and the blank control group were established. Following incubation for two days, total RNA was extracted using TRIzol™ reagent (Invitrogen Life Technologies). cDNA was synthesized using a ReverTra Dash^®^ RT-PCR kit (Toyobo, Osaka, Japan). Aliquots of total cDNA were amplified in a PC701 thermal cycler (Astec Co., Ltd., Fukuoka Japan). The reaction mixture was denatured at 94°C for 5 min, then subjected to 30 cycles of 94°C for 30 sec, 52°C for 50 sec and 72°C for 50 sec, followed by a final extension step at 72°C for 10 min. The amplification reaction products were resolved on 1.5% agarose/Tris-acetate-EDTA gels, electrophoresed at 100 mV and visualized via ethidium bromide staining. The gene expression of receptor activator of nuclear factor-κB ligand (RANKL) and osteoprotegerin (OPG) was quantified via normalization to the standard GAPDH (a routinely used reference gene). The primers used for the RT-PCR were as follows: RANKL upstream, 5′-TATGATGGAAGGCTCATGGT-3′ and downstream, 5′-TGTCCTGAACTTTGAAAGCC-3′; OPG upstream, 5′-AAAGCACCCTGTAGAAAACA-3′ and downstream, 5′-CCGTTTTATCCTCTCTA-3′; GAPDH upstream, 5′-TCCACTCACGGCAAATTCAACG-3′ and downstream, 5′-TAGACTCCACGACATACTCAGC-3′ (Invitrogen Life Technologies).

### ELISA

OCCM-30 cells at a density of 2×10^5^ were cultured in a six-well plate overnight. Following culture, the cells were washed twice with PBS for 10 sec and the three experimental groups (50 μg/ml LiCl, 25 ng/ml LPS and 50 μg/ml LiCl + 25 ng/ml LPS) and the blank control group were established. The experimental groups were treated for 48 h. Supernatants from the cell culture were harvested via centrifugation (150 × g, 4 min, room temperature) and stored at -20°C. The OPG levels in the supernatants and RANKL levels in the cell lysate were measured using mouse ELISA kits (R&D Systems Inc., Minneapolis, MN, USA) in accordance with the manufacturer’s instructions.

### Statistical analysis

Data are expressed as the mean ± standard deviation. The statistical analysis was performed using Origin 8.0 software (OriginLab Corp., Northampton, MA, USA).

## Results

### Cell viability analysis of cementoblasts

**Figure 1:**
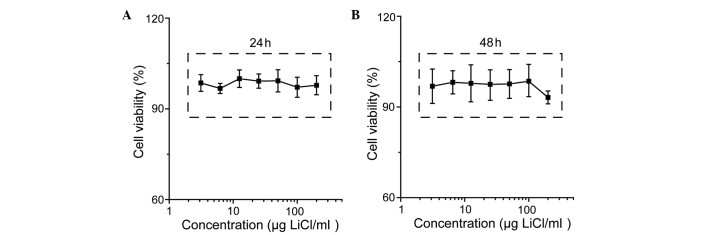
Effect of LiCl on the viability of OCCM-30 cells following incubation for (A) 24 h and (B) 48 h, as measured via MTT assay. Results are presented as the mean ± standard deviation. LiCl, lithium chloride.

**Figure 2:**
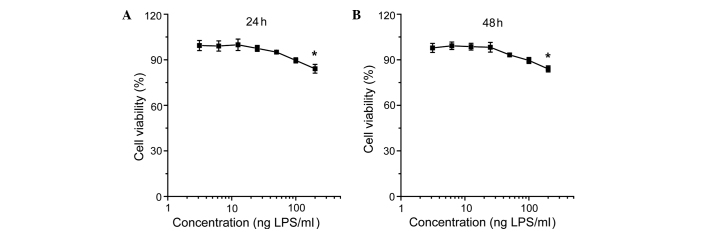
Effect of LPS on the viability of OCCM-30 cells following incubation for (A) 24 h and (B) 48 h, as measured via MTT assay. Results are presented as the mean ± standard deviation. ^*^P<0.05, as compared with the untreated cells, which were regarded as 100%. LPS, lipopolysaccharide.

**Figure 3:**
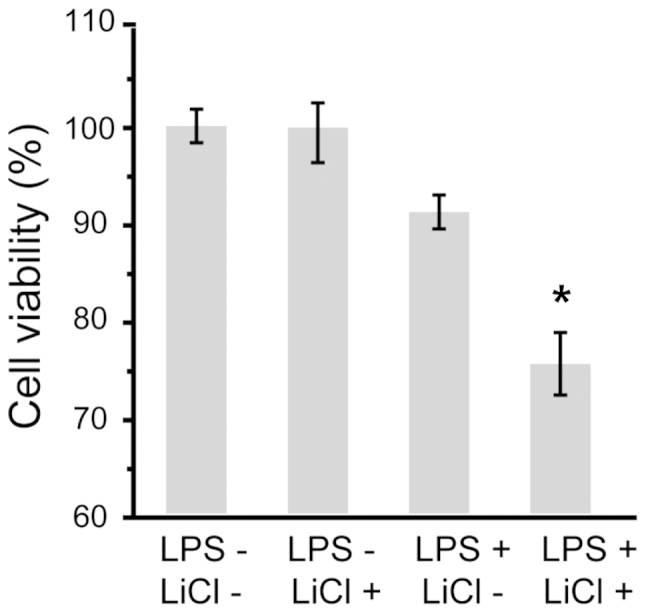
Cell viability evaluated via MTT assay. Results are presented as the mean ± standard deviation. ^*^P<0.05 vs. untreated cells. LPS, lipopolysaccharide; LiCl, lithium chloride.

As shown in Fig. 1, the viability of the cementoblasts was not significantly affected by LiCl; however, incubation with LPS reduced cell viability in a concentration-dependent manner (Fig. 2). The effect of inhibition was more evident for incubation with LiCl and LPS at the indicated concentrations (Fig. 3).

### Effect of LiCl on differentiation in the presence of LPS

**Figure 4:**
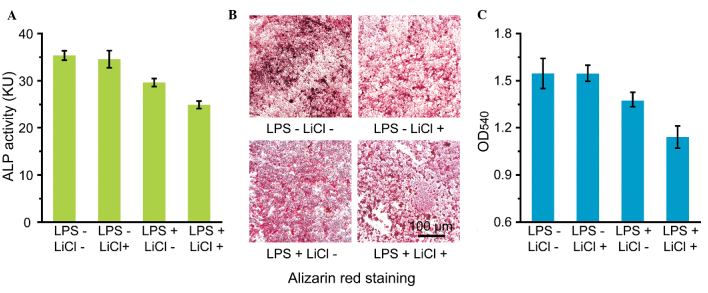
(A) ALP activity; (B) alizarin red staining images and (C) quantitative analysis of the staining of OCCM-30 cells. Results are presented as the mean ± standard deviation. ALP, alkaline phosphatase; LPS, lipopolysaccharide; LiCl, lithium chloride; OD, optical density.

As Fig. 4A shows, co-incubation with LiCl and LPS decreased the expression of ALP in the cementoblasts. Similarly, the mineralization of the cementoblasts under the co-incubation condition was markedly reduced compared with that of the control group (Fig. 4B and C).

### Analysis of OPG and RANKL expression

**Figure 5:**
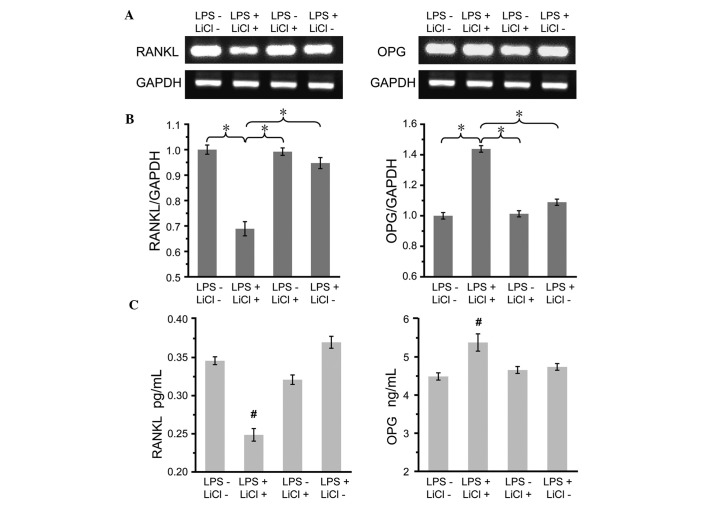
RANKL and OPG expression in OCCM-30 cells under various treatment conditions. (A) Reverse transcription-polymerase chain reaction results.(B) mRNA expression of RANKL and OPG quantified via normalization to the standard, GAPDH. (C) ELISA analysis of the associated proteins. ^*^P<0.05; ^#^P<0.05 vs. untreated cells. Results are presented as the mean ± standard deviation. LPS, lipopolysaccharide; LiCl, lithium chloride; RANKL, receptor activator of nuclear factor-κB; OPG, osteoprotegerin.

The mRNA expression of OPG and RANKL was firstly assessed under the condition of co-incubation. As shown in Fig. 5A and B, RANKL mRNA was expressed at low levels while OPG mRNA expression was markedly enhanced compared with that in the control group. Similar trends were observed in the RANKL and OPG protein expression, as determined using ELISA. In the co-incubation group, decreased RANKL and increased OPG protein expression was observed compared with the control group (Fig. 5C).

## Discussion

Periodontal tissue loss can be a frequent obstacle preventing successful treatment in patients with periodontitis. With the development of tissue engineering, a variety of scaffolds modified by different biological agents have been developed. Among these agents, lithium ions have been demonstrated to facilitate bone formation and have been used in the scaffold design process (3). In a previous study, we have shown that lithium ions can affect the metabolism of the cementum (23); however, in that study, cementum resorption was evaluated under normal conditions but not under an inflammatory state. The aim of the present study, therefore, was to establish a periodontitis model *in vitro* and evaluate the effect of lithium ions in the presence of LPS.

To the best of our knowledge, this study has been the first to evaluate the effect of lithium ions on a periodontitis model. Secreted by Gram-negative bacteria, LPS has been reported to trigger inflammatory responses and cause the destruction of periodontal tissues. In addition, LPS can stimulate osteoblasts to produce cytokines and receptor activators, inhibit osteoblast differentiation and affect gene expression (24–27). Based on this evidence, LPS was selected as the agent used to establish the periodontitis model in the present study. MTT assay revealed that incubation of cementoblasts with LiCl had little effect on the cementoblast survival (Fig. 1); however, LPS reduced cell viability in a concentration-dependent manner, demonstrating the negative effect of LPS on cell proliferation (Fig. 2). In accordance with the MTT results and the findings of previous studies (8,12,19), 50 μg/ml LiCl and 25 ng/ml LPS were selected as the incubation concentrations for the other experiments. As shown in Fig. 3, the inhibitory effect of LPS on cementoblast proliferation was significantly aggravated in the co-incubation condition. It has been well accepted that proliferation is an essential process for cementogenesis, particularly in its early stage. The effect of lithium ions on a periodontitis model could, therefore, provide novel insight into cementogenesis.

As an early marker of cementoblast differentiation, ALP plays an important role in transferring phosphate groups from the cells to the matrix. The incubation of cementoblasts with LiCl alone showed nearly no effect on the ALP activity of the cells, which was in accordance with the results of a previous report (Fig. 4A) (8). The activity of ALP was slightly reduced by LPS due to the negative effect of LPS on cell differentiation. By contrast, the ALP activity decreased markedly under the co-incubation condition, which was consistent with the results of the MTT assays. Alizarin red staining (Fig. 4B) and quantitative OD value analysis (Fig. 4C) further demonstrated the formation of red-stained mineralized nodules and the evident decrease in mineralization in the cementoblasts under the co-incubation condition, indicating that incubation with LiCl and LPS could reduce the ALP activity and further aggravate the negative regulatory effect on cementoblast differentiation.

Wnt/β-catenin signaling is involved in increases in bone mass via numerous mechanisms, including the induction of osteogenesis and stem cell renewal, which indicates that the activation of wnt signaling may facilitate the regeneration of bone and associated periodontal tissues (28). Previous findings have shown that LiCl can activate wnt signaling to suppress cementoblast functions (8). Performing an important role in osteoclast differentiation and bone resorption, RANKL can bind with its cognate RANK receptor on the surface of pre-osteoclasts and trigger differentiation towards mature osteoclasts. OPG can block the action of RANKL and protect bone from resorption. Generally, decreased RANKL or increased OPG expression can promote cementum formation and provide an osteoprotective condition (21); therefore, the effect of LiCl on the expression of OPG and RANKL in the presence of LPS was evaluated in the present study. Fig. 5A and B demonstrated that LiCl had the potential to alter osteoclastogenesis by regulating the OPG/RANKL ratio. In previous studies, exposure to LPS alone led to a slight enhancement in OPG and RANKL mRNA expression due to a self-protection reaction (22,29,30). In previous studies using osteoblasts (31,32), incubation with LiCl alone could upregulate OPG and downregulate RANKL mRNA expression. When the effect of LiCl was evaluated in the present periodontitis model, reduced RANKL mRNA and elevated OPG mRNA expression was observed. Additional investigations into the protein expression levels of OPG and RANKL revealed similar trends (Fig. 5C). The results in combination have demonstrated that LiCl may be beneficial to cementum protection via alleviating osteoclastogenesis.

The present study is the first, to the best of our knowledge, to evaluate the effect of lithium ions on a periodontitis model established using LPS. Negative effects were observed for cementoblast proliferation, while positive effects were noted for the inhibition of osteoclastogenesis. Detailed investigations, including MTT assays, microscopic imaging and the quantitative analysis of mRNA and protein expression, were performed, and the results indicated that bacterial infection should be controlled prior to the application of lithium ions for treatment. The present methodology could be extended to other disease-treatment systems by changing the disease model and materials, which would enable further applications associated with treatment and therapy in the clinic. In conclusion, lithium ions can be applied to facilitate cementum repair in scaffold design; however, its potential toxicity due to the presence of LPS warrant additional consideration.
